# Anaphylactic Shock After Cervical Conization Hemostasis With a Packing Soaked in Monsel’s Solution

**DOI:** 10.7759/cureus.51603

**Published:** 2024-01-03

**Authors:** Luís Pedro, Ana Gonçalves, Maria I Sousa, Sónia Duarte, Teresa Leal, Sandra Soares

**Affiliations:** 1 Anesthesiology and Critical Care, Centro Hospitalar Universitário de Santo António, Porto, PRT; 2 Obstetrics and Gynecology, Centro Hospitalar Universitário de Santo António, Porto, PRT; 3 Anesthesiology and Critical Care, Centro Hospitalar Universitário do Porto, Porto, PRT

**Keywords:** monsel's solution, perioperative care, cervical conization, shock, anaphylaxis

## Abstract

Anaphylactic shock is a life-threatening medical emergency, and its successful approach depends on early recognition and treatment. We present a case report of a 54-year-old female, with the American Society of Anesthesiology (ASA) Physical Status Classification III, admitted for cervical conization. She presented with known allergies to paracetamol, diclofenac, and nimesulide, and a history of nickel contact dermatitis, with no reports of complicated anesthesia. During conization, adrenaline was infiltrated in the cervix, and hemostasis was performed with packing soaked in Monsel’s solution. The immediate postoperative period in the post-anesthesia care unit was uneventful, and no drugs were administered during this period. Three hours after discharge to the ward, the patient had progressive dyspnea with desaturation and maculopapular exanthema. Anesthesia medical emergency was activated. Upon arrival of the emergency team, the patient presented: marked edema of the lips and tongue, respiratory distress, SpO_2_ 82% (under non-rebreathing high concentration oxygen mask), audible vesicular murmur but diminished in all lung fields (without bronchospasm), blood pressure of 60/40 mmHg, increased capillary refill time (4-5 seconds), Glasgow Coma Scale score of 14, as well as generalized maculopapular exanthema and eyelid edema. Gas analysis revealed the following: pH 7.36, pO_2_ 150, pCO_2_ 33, HCO_3_ 22, and lactate 2.2 mmol/L. Anaphylactic shock was immediately diagnosed without an identified causative agent. Intramuscular adrenaline (0.5mg), endovenous hydrocortisone (200 mg), clemastine (2 mg), and profuse fluid therapy were administered. There was an initial slight improvement followed by subsequent worsening. Additional administration of 0.5 mg intramuscular adrenaline and endovenous methylprednisolone (125 mg) provided similar results. Considering that no other drugs were administered in the ward, the emergency team and the attending gynecologist assumed an association between nickel allergy and the chemical composition of Monsel’s solute. Thus, it was decided to remove the packing soaked in Monsel’s solute from the vaginal cavity and wash it with saline solution. After removing the packing and further administration of 0.5 mg intramuscular adrenaline, there was progressive improvement in the blood pressure and SpO_2_. Tryptase samples collected one hour later were increased (23.9 ug/L; normal: <11.4 ug/L). The patient was shifted to the intensive care unit for surveillance, from which she was discharged after 2 days, with scheduled immunoallergology consultation, which is waiting.

This case highlights the importance of causative agent identification as a key point for anaphylactic shock resolution, as well as a multidisciplinary discussion among professionals.

## Introduction

Perioperative anaphylaxis refers to a severe and potentially life-threatening allergic reaction that occurs in response to exposure to substances used during the perioperative period, which includes the time before, during, and after a surgical procedure. While any substance can cause perioperative anaphylaxis, the most common culprits in the anesthetic theater include antibiotics, neuromuscular blocking agents, disinfectants, and latex, although these may vary based on geographic location [1]. In some cases, the cause is not obvious (idiopathic anaphylaxis), and investigations for rarer allergens or differential diagnoses should be considered.

The symptoms of anaphylaxis are highly variable. Data from patients experiencing anaphylaxis indicate that skin and mucosal symptoms occur most frequently (>90% of cases), followed by symptoms involving the respiratory and cardiovascular systems (>50%) [2].

Managing perioperative anaphylaxis requires swift and coordinated action by the multidisciplinary medical team. It is essential to discontinue the administration of the suspected trigger, administer adrenaline to counteract severe symptoms, provide respiratory support if needed, and stabilize blood pressure. Subsequent investigation into the trigger of the reaction can involve allergy testing and thorough documentation to prevent future exposures.

Monsel's solution is a topical hemostatic agent based on ferrous sulfate, which can be applied directly to the surgical site after lesion excision. It causes agglutination of surface proteins, resulting in hemostasis. The application is quite safe, and the most commonly described adverse effect is hyperpigmentation at the application site; there are no cases of anaphylaxis published in the literature [3].

We report a case of anaphylaxis induced by Monsel's solution, specifically focusing on its recognition and management.

This article was previously presented as a poster at the "Congresso da Sociedade Portuguesa de Anestesiologia 2023 - Empower Yourself" on March 25, 2023.

## Case presentation

A 54-year-old female, with the diagnosis of carcinoma in situ of the cervix, American Society of Anesthesiology (ASA) Physical Status III, was electively admitted for cervical conization procedure. She had a past medical history of smoking, fibromyalgia, atrial fibrillation, and was a pacemaker carrier (due to bradycardia with repetitive syncope). Allergies to paracetamol, diclofenac, and nimesulide were known, with symptoms of urticaria and dyspnea in provocation test and a history of nickel contact dermatitis, but there were no reports of complications during previous anesthesia procedures.

The cervical conization procedure was successful and uneventful under monitored anesthesia care with fentanyl and propofol, along with analgesia provided by tramadol. During the conization, adrenaline was infiltrated into the cervix, and hemostasis was achieved using Spongostan soaked in Monsel's solution. The immediate postoperative period in the postanesthesia care unit (PACU) was uneventful, and no drugs were administered during this time. The patient was then discharged two hours later to the ward. However, one hour after discharge, she reported progressive dyspnea and desaturation and developed a maculopapular exanthema. An anesthesia medical emergency was activated.

Upon arrival of the emergency team, the patient presented: marked edema of the lips and tongue, respiratory distress, SpO_2_ 82% (under non-rebreathing high concentration oxygen mask), audible vesicular murmur but diminished in all lung fields (without bronchospasm), blood pressure of 60/40 mmHg, increased capillary refill time (4-5 seconds), Glasgow Coma Scale score of 14, as well as generalized maculopapular exanthema and eyelid edema. Gas analysis revealed the following: pH 7.36, pO_2_ 150, pCO_2_ 33, HCO_3_ 22, and lactate 2.2 mmol/L.

Anaphylactic shock was immediately diagnosed without an identified causative agent. This led to the administration of 0.5 mg adrenaline intramuscularly, and 200 mg hydrocortisone and 2 mg clemastine, both intravenously. Additionally, leg elevation and a crystalloid bolus were administered. Although there was initial slight improvement, subsequent worsening occurred. Additionally, 0.5 mg adrenaline intramuscularly and 125 mg methylprednisolone intravenously were administered with similar results.

Considering that no new drugs were administered in the ward, the emergency team and attending gynecologist suspected an association between nickel allergy and the chemical composition of Monsel's solution, which was used for hemostasis after the surgery. As a result, it was decided to remove the packing soaked in Monsel's solution from the vaginal cavity and wash it with saline solution. Following the removal of the solution-soaked packing and an additional administration of 0.5mg adrenaline intramuscularly, there was a progressive improvement in blood pressure and SpO_2_ levels. Tryptase sample collected 1 hour later was increased to 23.9 µg/L (normal range: <11.4 µg/L), and basal tryptase level after 24 hours was 6.38 µg/L.

The patient was transferred to the intensive care unit for surveillance. She was discharged after two days with a recommendation to complete a five-day course of oral corticosteroids (prednisolone 20 mg twice daily), undergo a follow-up analytical study within one week, and have an evaluation by an immunologist/allergist.

## Discussion

Rapid recognition and avoidance of the causative agent are critical in the management of patients with anaphylactic shock. It is also important to promptly treat anaphylaxis as it seems to be more responsive to treatment in its early phases. This is based on the observation that delayed adrenaline injection is associated with fatalities [4].

In this case, identifying the causative agent was difficult due to the time interval since the last drug administration, possibly because of cervical adrenaline infiltration. The initial clinical improvement achieved with the administration of intramuscular adrenaline was followed by a worsening of the condition, which alerted the team to the possibility of the causative agent's persistence. After discussing the case between the gynecology and anesthesia emergency teams and considering the patient's allergy history, particularly regarding nickel contact dermatitis, it became possible to identify Monsel's solution as the causative agent. When specific materials are used, professional discussion is critical for correctly interpreting and resolving the case. This case demonstrates that identifying the causative agent and eliminating it are key points for successful resolution of an anaphylactic shock.

Serum tryptase levels may help support the diagnosis during later allergy consultations. The European Academy of Allergy and Clinical Immunology task force suggests measuring serum tryptase levels half an hour to two hours after the reaction begins (23.9 mcg/L) and measuring baseline tryptase at least 24 hours after complete symptom resolution (baseline tryptase was 6.38 mcg/L) to aid in the diagnosis of anaphylaxis, respectively [2].

Cases of recurrent anaphylactic symptoms have been reported, occurring after an asymptomatic period longer than one hour and lasting up to 72 hours. This phenomenon is known as biphasic anaphylaxis. Given this, it is essential to admit patients to units with continuous surveillance to identify symptoms and intervene early [5].

## Conclusions

The continuous advancement in the fields of medicine and industry gives rise to the introduction of novel substances characterized by specific properties deemed valuable across diverse domains. In the context of this clinical scenario, Monsel's solution, extensively employed in gynecology for its hemostatic attributes, emerged as the probable causative agent of anaphylaxis, as evidenced by the resolution of symptoms following its removal. The unfamiliarity of the internal emergency team with the surgical technique and substances implemented underscored the imperative of interdisciplinary discourse to conclusively identify the agent responsible for the anaphylactic shock.
